# Relationship Between Cognitive Function and Balance, Fatigue, and Physical Activity Levels in Patients with Liver Cirrhosis

**DOI:** 10.3390/healthcare14050643

**Published:** 2026-03-04

**Authors:** İlker Demir, Bilsev Demir

**Affiliations:** 1 Department of Physical Medicine and Rehabilitation, Turgut Ozal Medical Center, İnonu University, Malatya 44280, Turkey; fztilkerdemir@gmail.com; 2 Division of Surgical Nursing, Nursing Department, Faculty of Health Sciences, Malatya Turgut Ozal University, Malatya 44900, Turkey

**Keywords:** liver cirrhosis, cognitive function, balance, physical activity, fatigue

## Abstract

**Background**: Liver cirrhosis is a chronic and progressive disease, and it affects liver parenchymal cells. Although it is known that during the course of this disease, cognitive function, balance, and physical activity levels decrease, and fatigue severity increases, the relationship between these variables remains unclear. This study is the first to examine the relationship between cognitive function level and balance, fatigue, and physical activity levels in patients with liver cirrhosis and was conducted to provide a new perspective on treatment. **Aim**: This study aims to investigate the relationship between cognitive function and balance, physical activity, and fatigue in patients with liver cirrhosis. **Method**: A total of 132 patients were included in the study. Cognitive function levels of the patients were measured with the Montreal Cognitive Assessment Scale, balance performance using the One-Legged Stance Test and timed up and go tests, physical activity levels with the International Physical Activity Scale-Short Form, and fatigue levels with the Fatigue Severity Scale. **Results**: Correlation analyses showed that cognitive function (MoCA) was significantly associated with static balance (r = 0.232, *p* = 0.007) and fatigue severity (r = −0.297, *p* = 0.001), whereas no statistically significant relationships were observed with dynamic balance (r = −0.068, *p* = 0.441) or physical activity (r = −0.011, *p* = 0.903). Multivariable regression analyses indicated that disease duration (β = 0.02, *p* = 0.009) and exercise habits (β = 0.65, *p* = 0.031) were independently associated with cognitive function (MoCA), while disease duration was also independently associated with static balance performance (β = 0.08, *p* = 0.002). **Conclusions**: These findings indicate statistically significant associations between cognitive function, static balance, and fatigue severity, whereas no significant associations were observed with dynamic balance or physical activity. These relationships should be interpreted as associative rather than causal and suggest that cognitive status may be clinically relevant when evaluating balance performance and fatigue burden in patients with cirrhosis.

## 1. Introduction

Liver cirrhosis is a chronic and progressive disease that occurs due to viral infection or alcohol use. It is characterized by widespread fibrosis and hepatocellular necrosis. As the disease progresses, it turns into decompensated cirrhosis, and symptoms such as jaundice emerge. Liver failure may occur due to cirrhosis. Consequently, hepatic function is compromised, resulting in elevated levels of numerous compounds in the blood. Manganese and ammonia levels are the most important among these compounds for the central nervous system. In liver cirrhosis, impaired hepatic clearance can result in the build-up of neurotoxic substances, such as ammonia and manganese. These metabolic disturbances have been linked to changes in brain function, particularly in cortico–subcortical networks. However, the current evidence base indicates that cognitive and motor impairments in cirrhosis have multiple causes and cannot be attributed to a single neurotoxic mechanism [1]. Cirrhosis is associated with a decline in cognitive function.

In addition to cognitive function impairment, many physical problems, such as gait disorders, autonomic dysfunction, sleep disorders, loss of muscle strength, and peripheral and central fatigue, are observed in patients with cirrhosis. Additionally, frailty may be observed due to a decrease in physiological reserves and a decline in functional status [2]. Frailty is a condition that arises from impaired motor and cognitive functions, that is, impaired physical and mental vitality. Recently, it can occur in patients with impaired liver function, regardless of age [3]. It has been reported that the average age of liver cirrhosis patients with frailty is 59 years, and 40 percent of cirrhosis patients are functionally impaired, with one in five patients considered frail [2,4]. The effects of frailty, which are readily apparent in daily clinical practice, include falls, fractures, hospital admissions, limited recovery from injuries, and even death [2]. Patients experience increased fatigue severity, and their balance and physical activity levels decrease [5,6,7]. The aforementioned issues have a detrimental effect on the day-to-day activities of individuals [8]. A review of the literature reveals that, although the physical and cognitive problems observed in cirrhosis were revealed by research, no study was found examining the effects of patients’ cognitive function levels on physical performance. In order to create effective exercise programs for diseases, it is important to determine the issues and their interrelationships. These clinical issues can exacerbate one another, creating a cycle. In order to achieve recovery, a holistic approach should be created by addressing physical and cognitive problems together. In this study, the relationship between cognitive function level and balance, physical activity, and fatigue levels was examined in order to determine the relationship between cognitive and physical function levels in cirrhosis patients. We believe that the data obtained will contribute to determining priorities and creating a program when creating an exercise program.

## 2. Materials and Methods

### 2.1. Study Design and Sample

**Figure 1:**
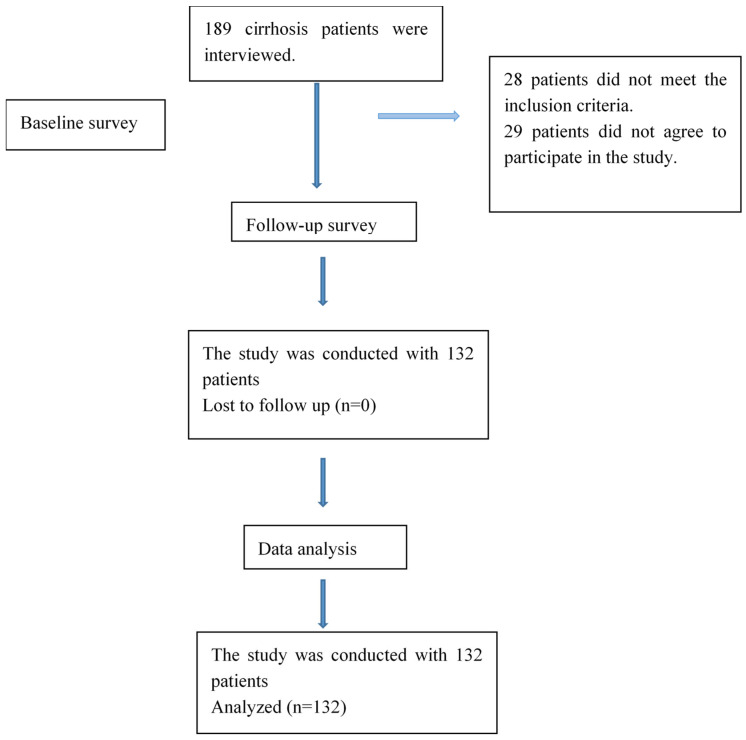
Flowchart of the study design.

In this study, we investigated the relationship between cognitive function and balance, physical activity, and fatigue in patients with liver cirrhosis. The research flow chart is shown in Figure 1. This is a descriptive study with a correlational design. Data collection was conducted between January and May 2025. The data collection process was conducted by researchers at the organ transplantation institute of a university hospital located in eastern Turkey. Evaluations were conducted on each patient in a face-to-face manner within a hospital environment by the same physiotherapist. The sample consisted of liver cirrhosis patients who met the inclusion and exclusion criteria. An a priori sample size calculation was performed using G*Power software (version 3.1.9.7; Heinrich-Heine-Universität Düsseldorf, Düsseldorf, Germany; https://www.psychologie.hhu.de/arbeitsgruppen/allgemeine-psychologie-und-arbeitspsychologie/gpower (accessed on 26 February 2026)), based on a two-tailed bivariate correlation model, since the primary objective was to examine the association between cognitive and physical parameters. Assuming a medium effect size (r = 0.30), an alpha level of 0.05, and a statistical power of 0.95, the minimum required sample size was calculated to be around 130 participants. To compensate for potential exclusions, 189 cirrhosis patients were interviewed. After excluding twenty-eight patients who did not meet the inclusion criteria and twenty-nine who declined to participate, the final sample comprised 132 patients. (Figure 1). The inclusion and exclusion criteria for the sample are shown below:

### 2.2. Inclusion and Exclusion Criteria

The inclusion criteria for this study were (i) individuals diagnosed with cirrhosis by clinical, ultrasonographic, and/or liver biopsy; (ii) patients with liver cirrhosis were offered participation in the study during routine outpatient follow-up and accepted the offer; (iii) hemodynamically stable; (iv) aged 18 years and over, conscious, cooperative, and able to communicate; and (v) not being a participant in another study where a similar initiative was being conducted at the same time. Failure to meet these criteria was accepted as the exclusion criterion.

### 2.3. Data Collection Tools

The data collection tools administered to patients with liver cirrhosis included a demographic information form, the Montreal Cognitive Assessment Scale, the Timed Up and Go Test, the One-legged Stance Test, and the International Physical Activity Questionnaire Short Form (Short Form IPAQ). Information regarding the data collection tools is provided below.

### 2.4. Demographic Information Form

The demographic information form was developed by researchers based on the literature. The patients were asked about their age, gender, educational level, body mass index, employment status, marital status, disease duration, cirrhosis type, exercise habits, smoking, and alcohol use.

### 2.5. The Montreal Cognitive Assessment Scale (MoCA)

The Montreal Cognitive Assessment Scale was developed by Nasreddine et al. (2005) [9] and is utilized to evaluate cognitive functions in individuals. The Turkish validity study of this scale was conducted by Selekler et al. [6]. Cognitive parameters such as attention, concentration, memory, executive functions, and visual–spatial skills are evaluated with this scale. Although the MoCA includes items related to different cognitive domains, it is primarily intended to provide a global cognitive score. Domain-specific subscale scores are not independently validated, so only the total score was used in the analyses. The test score ranges from 0 to 30 points, with the assessment focusing on executive functions and abstract thinking, as evidenced by tasks such as drawing a clock. For a result to be evaluated as indicative of a cognitive disorder, a score of 26 and above is generally required to indicate normal cognitive functioning. Scores of 21 and above may indicate performance close to the normal range [9,10]. The Cronbach’s alpha internal consistency coefficient for this study was found to be 0.58.

### 2.6. Timed Up and Go Test

The Timed Up and Go (TUG) test was utilized to evaluate functional mobility and dynamic balance. Participants were instructed to rise from a standard chair with armrests, traverse a distance of three meters at a pace deemed to be comfortable and safe, effect a turn, and then return to the chair and sit down once more. The duration of the task was measured in seconds using a stopwatch. It was hypothesized that lower completion times would be indicative of superior functional mobility and dynamic balance performance.

### 2.7. One-Legged Stance Test

The test was performed to measure static standing capacity. Before starting the test, the patient was asked about their dominant extremity or which foot they wanted to stand on. First, the person was asked to put equal weight on both lower extremities, and in this case, with their eyes open, they were asked to lift one foot without touching the supporting leg. The time started when the person lifted their foot. The time was stopped when the patient’s raised foot touched the ground, jumped on the supporting leg, or received support to maintain their balance. The test was performed twice with eyes open, and the average time was recorded. Higher values reflected better static balance performance [11].

### 2.8. Fatigue Severity Scale (FSS)

The Fatigue Severity Scale was developed by Krupp et al. (1989) [12] and is used to assess the fatigue level of adults. The Fatigue Severity Scale was adapted into Turkish by Armutlu et al. (2007) [13]. This scale consists of a total of 9 questions. The lowest score that can be obtained from the scale is 9, and the highest score is 63. The scale scores are the average value of nine sections. When calculating the score based on the answers to the questions, patients with a score of less than 4 are considered ‘not tired’, and those with a score of 4 or more are considered ‘tired’. The Cronbach’s alpha internal consistency coefficient for the original scale was found to be 0.94 [12,13]. The Cronbach’s alpha internal consistency coefficient for this study was found to be 0.78.

### 2.9. International Physical Activity Questionnaire Short Form (Short Form IPAQ)

The IPAQ was developed by Craig et al. (2003) [14] and is used to assess the physical activity level of adults. The IPAQ was adapted into Turkish by Bozkuş et al. (2013) [15]. The IPAQ consists of 7 items, and it has no subscales. The IPAQ respondents are asked to report the number of days and the duration of the vigorous (V), moderate (M), and walking activity (W), and a combined total physical activity score. The MET method was used to determine physical activity levels. MET stands for metabolic equivalent of task. Standard MET values were established for these activities. The established MET values are expressed as follows: Vigorous Physical Activity: 8.0 METs, Moderate Physical Activity: 4.0 METs, Walking Physical Activity: 3.3 METs, Sitting: 1.5 METs. The total energy expenditure of the participants was calculated as MET·min/week and analyzed as a continuous variable. This was done because higher values indicate higher levels of physical activity [14,15].

### 2.10. Data Analysis

The evaluation of the individuals participating in the study was carried out using the Windows-based IBM Statistical Package for Social Sciences (SPSS) 20.0 statistics program (Armonk, NY, USA). During the evaluation process of the data from the study, the following descriptive statistics were presented: mean, standard deviation, maximum, minimum, and percentile. The Shapiro–Wilk test was utilized in order to execute the requisite normality tests. In addition to bivariate analyses, multivariable linear regression models were constructed to examine the independent associations between cognitive function, balance, and fatigue outcomes. Separate models were created for the Montreal Cognitive Assessment (MoCA), the One-Legged Stance Test, and the Fatigue Severity Scale (FSS). All models were adjusted for age, sex, educational level, body mass index, disease duration, and exercise habits. The results were evaluated at a 95% confidence interval and *p* < 0.05.

### 2.11. Ethical Considerations

Before the start of this study, institutional approval was obtained from the Turgut Ozal Medical Center Liver Transplant Institute. Subsequently, Ethical Committee approval was obtained from the non-interventional ethics committee of Inonu University (Date: 03 January 2025, Decision No: 2025/6934). The purpose and details of the study were explained to the individuals who accepted to participate in the study in written and verbal form, and signed consent forms were obtained from the individuals, taking into account compliance with the Helsinki Declaration.

## 3. Results

**Figure 2:**
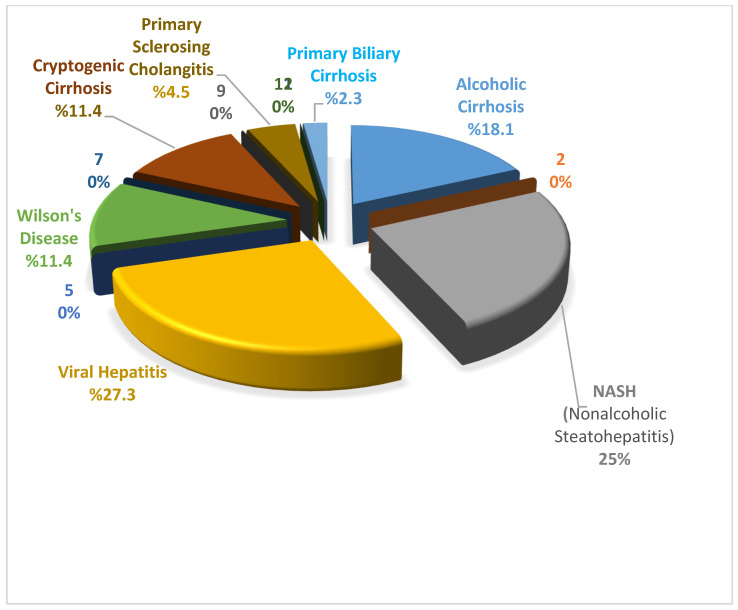
Distribution of cirrhosis etiology in the study population.

Table 1 shows the results of descriptive tests regarding the personal characteristics of liver cirrhosis. No statistically significant differences were found regarding gender, education, marital status, economic status, smoking, and alcohol use with cognitive assessment, fatigue, and physical activity (*p* > 0.05). Statistically significant differences were observed in terms of cognitive assessment, fatigue severity, and physical activity with exercise habits (*p* < 0.05). However, the highest prevalence is viral hepatitis, with 27.3%, while nonalcoholic steatohepatitis (NASH) is in second place with 25% (Figure 2).

Table 2 shows the results of mean scores for liver cirrhosis patients’ cognitive function, static and dynamic balance, fatigue, and physical activity. The patients’ cognitive functions were found to be at a moderate level. The One-Legged Stance Test, used to measure static balance, and the Timed Up and Go Test, used to measure dynamic balance, were 15.63 ± 5.39 and 10.36 ± 1.61, respectively. It was observed with decreased fatigue scores in patients and that the physical activity level of the patients was low.

Table 3 shows a correlation of the liver cirrhosis patients’ correlations among cognitive function, static balance, dynamic balance, fatigue severity, and physical activity. As shown in Table 3, a positive and significant relationship was found between the cognitive function and the one-legged stance test applied for measuring static balance (*p* < 0.01). At the same time, a significant negative relationship was found between cognitive function and fatigue severity (*p* < 0.01). No correlation was found between cognitive function level and physical activity and dynamic balance levels (*p* > 0.01).

Table 4 shows the results of the multivariable linear regression analyses investigating the relationship between cognitive function (MoCA), static balance (One-Legged Stance Test), the severity of fatigue (FSS), and physical activity level, after adjustment for age, sex, and disease duration. After adjusting for potential confounding factors, fatigue severity and static balance were found to be significant independent predictors of cognitive function. Specifically, higher fatigue levels were associated with poorer cognitive performance, while better static balance was associated with higher MoCA scores. Disease duration also showed an independent association with cognitive function. In contrast, physical activity, age, and sex were not significantly related to cognitive performance in the adjusted model. The magnitude of the standardized regression coefficients indicates that fatigue severity was the strongest predictor among the examined variables. These findings represent associative relationships identified within a cross-sectional framework and should not be interpreted as evidence of causality.

**Figure 3:**
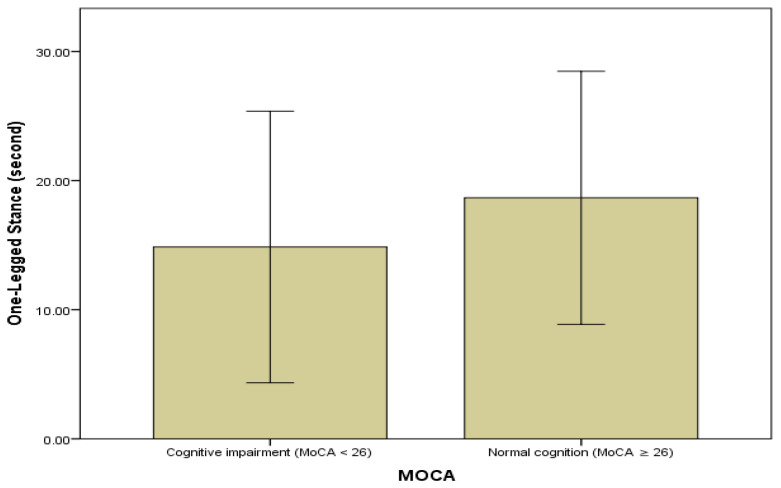
Association between Cognitive Function and Static Balance of Liver Cirrhosis Patients.

Figure 3 shows static balance performance according to cognitive function groups in liver cirrhosis patients. Patients with MoCA scores ≥ 24 demonstrated better static balance performance compared with those with MoCA scores < 24. For graphical presentation, MoCA scores were categorized using a cut-off value of 24 points.

**Figure 4:**
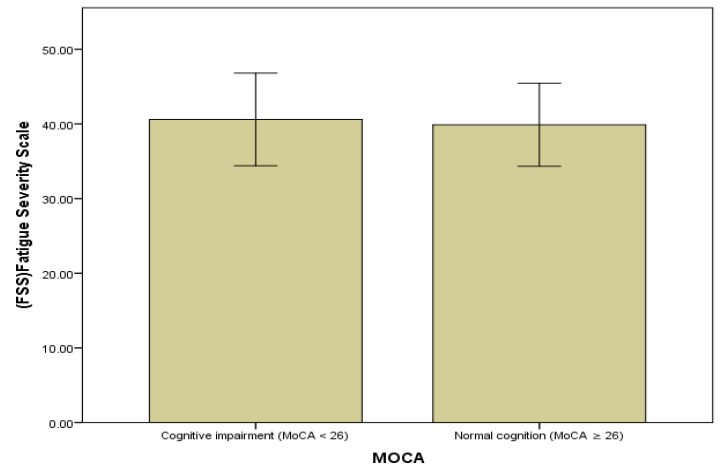
Association between cognitive function and fatigue severity of liver cirrhosis patients.

Figure 4 presents fatigue severity according to cognitive function groups in patients with liver cirrhosis. Patients with MoCA scores < 24 exhibited higher fatigue severity compared with those with MoCA scores ≥ 24. For graphical presentation, MoCA scores were categorized using a cut-off value of 24 points.

## 4. Discussion

This study investigated whether fatigue severity, static balance performance, and physical activity level predict cognitive function in patients with liver cirrhosis. Using a multivariable linear regression model adjusted for age, sex, and disease duration, we found that fatigue severity and static balance performance were significant independent predictors of cognitive function. Specifically, higher fatigue levels were associated with lower Montreal Cognitive Assessment (MoCA) scores, whereas better static balance performance was positively associated with cognitive function. Disease duration also showed an independent association with cognitive performance; however, physical activity was not significantly related to cognitive function in the adjusted model. These findings emphasize the close relationship between cognitive and physical health in patients with cirrhosis, and highlight the importance of assessing fatigue and balance when evaluating cognitive function.

Cirrhosis is a diffuse, chronic, and progressive disease characterized by the destruction of liver parenchymal cells and the development of widespread fibrosis. Symptoms of the disease include muscle cramps, dyspnea, tachypnea, weakness, anorexia, edema, fever of unknown origin, and sexual dysfunction [16,17,18]. When the liver does not perform its function, substances such as ammonia and manganese accumulate in the systemic circulation and pass through the blood–brain barrier, potentially contributing to cerebral damage and cognitive function loss. The balance impairment experienced by patients with cirrhosis is likely to be influenced by a combination of factors, such as muscle weakness, sarcopenia, altered proprioception, fatigue, and subtle cognitive dysfunction, rather than by a single, direct metabolic effect [19]. Although its pathogenesis cannot be fully explained, fatigue is one of the common symptoms in cirrhosis, and it is reported that fatigue severity decreases after liver transplantation [20]. Several studies examining cognitive–motor interactions have been conducted in non-cirrhotic populations, such as older adults or healthy individuals. While these findings provide indirect insight into potential mechanisms, they are not directly applicable to patients with cirrhosis due to disease-specific metabolic and neuromuscular alterations. Therefore, extrapolating these findings to cirrhotic populations should be approached with caution. By contrast, the present study focuses specifically on patients with cirrhosis, highlighting the associations observed in this clinical context and emphasizing the need for research into cirrhosis in this area. Recent contributions to the field of hepatology have indicated the presence of trace element accumulation, including manganese deposition, within basal ganglia structures in a subset of patients diagnosed with cirrhosis. However, current evidence supports interpreting these findings as associative rather than causal, and manganese accumulation is considered one of several physiological processes that may coexist with cognitive and motor alterations. Other interacting mechanisms, such as inflammation, metabolic imbalance, sarcopenia, and neurochemical alterations, may also contribute. Therefore, these findings should be evaluated within a multifactorial framework rather than attributed to a single determinant.

In order to develop an effective exercise program to reduce the adverse effects of cirrhosis, it is first necessary to identify the underlying problems, determine the relationships between them, and then create a program that is tailored to meet the specific needs of the target group. The objective of the present study was twofold: firstly, to obtain new information about the relationship between cognitive function level and balance, fatigue, and physical activity levels in cirrhosis patients; and secondly, to contribute to the enrichment of existing scientific evidence. Therefore, the relationship between cognitive function level and balance, fatigue, and physical activity level was investigated. The study data showed that there was a positive correlation between cognitive function level and the one-legged stance test used to measure static balance and a negative correlation with the fatigue severity scale. However, no correlation was found between cognitive function level and dynamic balance and physical activity levels. Although it is known that there is a decrease in cognitive and physical capacities in patients with liver cirrhosis, no study was found that examines the relationship between cognitive function, balance, fatigue, and physical activity level [21]. Although this prevents the possibility of comparing the study with data from cirrhosis patients, we believe that it is important in terms of the originality of the study.

In our study, 59.1% of the patients were male, 72.7% were married, 31.1% smoked, 6.8% drank alcohol, and 17.6% exercised regularly. Only 11.4% exercised regularly (Table 1). A study by Huet et al. on patients with cirrhosis found that most patients smoked or drank alcohol [22]. The prevalence in our study was lower. We believe this discrepancy may be attributable to variations in religious beliefs and cultural backgrounds.

Recent studies on chronic liver diseases and cirrhosis have highlighted the high incidence of falls and fall-related injuries and the fact that falls are considered a serious cause of mortality and morbidity. Roman et al., in their study of 118 cirrhosis patients, found that 20.3% of patients had fallen within the past year, with a fracture rate of 29.1% [23]. Several studies have been conducted in the relevant literature, examining the relationship between cognitive function and balance. However, these studies have not focused on patients with cirrhosis. A study was conducted with geriatric individuals to examine the relationship between cognitive function and tests used to determine static and dynamic balance. The study concluded that cognitive impairment resulted in a reduction of static balance, yet did not induce any alterations in walking behavior [24]. Furthermore, studies have reported that cognitive tasks increased balance [25,26]. Amodio et al., in their study of 36 patients with cirrhosis, revealed psychomotor slowing and attributed this to dysfunction in the prefrontal cortex-basal ganglia pathways. The study also noted that although no impairment occurred in the posterior attention system in the early stages of hepatic encephalopathy, dysfunction of the anterior attention system may be more responsible for the impact on attention ability [27]. A key finding of the present study is the differential association between cognitive function and balance outcomes. Cognitive function was associated with static balance performance. However, no significant association was observed with dynamic balance measures. Static balance tasks rely heavily on sustained attention, executive control, and sensory integration, all of which may be subtly impaired in patients with cirrhosis, even when hepatic encephalopathy is not overtly present. In contrast, dynamic balance is influenced by a broader range of factors, including muscle strength, gait mechanics, cardiovascular capacity, and compensatory motor strategies. These factors may mask the contribution of cognitive function in cross-sectional analyses. Our data revealed a positive correlation between cognitive function and static balance in cirrhotic patients, but no correlation was found with dynamic balance. Based on these results, we believe that cognitive tasks should be included in the development of an exercise program focused on static balance in cirrhotic patients. Therefore, the absence of an association between cognitive function and dynamic balance in this cohort does not necessarily indicate an absence of interaction but may instead reflect the multifactorial nature of dynamic postural control in cirrhosis.

It is reported that fatigue functions as a mediator between objective and subjective cognition, with heightened fatigue levels and impaired cognitive abilities being concomitantly associated. Studies show that cognitive function and fatigue levels are negatively correlated [25,26,27]. This study examined the relationship between cognitive function and fatigue levels in cirrhosis patients and found a negative correlation between them. This finding aligns with existing literature on the subject, suggesting that the level of mental fatigue experienced by the participants may have contributed to the observed outcomes.

The relationship between physical activity and cognition began to be investigated with the pioneering study conducted by Spirdusa in 1975, and different studies were presented on this subject [22,28,29]. Studies generally show that physically active individuals exhibit better cognitive function than their inactive peers. Greater participation is reported to be associated with better cognitive function [22,30,31]. In addition to the aforementioned studies, others have been conducted that report no significant effect of cardiovascular fitness on any aspect of cognition [32]. Based on the data we obtained, no relationship was found between cognitive function levels and physical activity levels in cirrhosis patients. This result is parallel to the prevailing view in the literature. The lack of association between cognitive function and physical activity observed in our study may reflect the complex and heterogeneous nature of cognitive impairment in cirrhosis. Despite the established correlation between metabolic factors, including ammonia and manganese accumulation, and cognitive dysfunction, these factors alone are unlikely to fully account for behavioral outcomes such as physical activity. The lack of an independent association between cognitive function and physical activity requires careful consideration. While neurotoxic mechanisms related to cirrhosis may contribute to cognitive impairment, alternative explanations should also be considered. Low overall levels of physical activity in this population, disease severity, the burden of fatigue, and physical deconditioning may limit variability in activity measures, thereby reducing the ability to detect associations in cross-sectional analyses. Furthermore, the physical activity behavior of patients with cirrhosis is likely influenced by multiple psychosocial and clinical factors beyond cognitive status alone.

From a clinical perspective, these findings have potential implications for the design of rehabilitation and exercise programs for patients with cirrhosis. The observed associations between cognitive function, static balance, and fatigue imply that cognitive status should be considered when planning balance-oriented interventions. Incorporating cognitive screening and targeted balance training could help to identify patients at a higher risk of functional impairment. Furthermore, personalized exercise programs that consider fatigue severity and cognitive limitations could improve adherence and functional outcomes. Further interventional studies are required to ascertain whether integrated cognitive–motor rehabilitation strategies can improve balance and quality of life in this patient group. Several potential confounding factors should be considered when interpreting these findings. These include age, comorbid conditions, medication use, nutritional status, and a history of hepatic encephalopathy, all of which may influence cognitive performance, balance, fatigue, and physical activity levels in patients with cirrhosis. While age was included in the multivariable models, the other factors were not systematically evaluated and consequently could not be adjusted for. These unmeasured variables may contribute to residual confounding and should be addressed in future studies with a more comprehensive clinical characterization.

## 5. Limitations

It is acknowledged that cognitive function, balance, physical activity, and fatigue levels are impacted in individuals suffering from cirrhosis. However, no research has been conducted to examine the interrelationship between these factors. This study represents a pioneering effort in this field. In this respect, it is hypothesized that the present study will serve as a resource for future research. The internal consistency of the MoCA was moderate in this sample (α = 0.58). This may affect the accuracy of cognitive measurements and should be taken into account when interpreting the results. First, the relatively small and heterogeneous sample size may limit the generalizability of the findings. Given the absence of prior research in this area, the data could not be compared with the results of any other studies. The length of hospital treatment limited the possibility of longer-term treatment and follow-up. The number of patients and their cultural diversity were limited because the study was conducted at a single center. Therefore, we believe that studies conducted in different regions, involving multiple healthcare institutions and a larger number of participants, would contribute to the literature. Second, several clinically relevant factors that may influence cognitive function, balance, physical activity, and fatigue—such as comorbidities, medication use, nutritional status, and a history of hepatic encephalopathy, disease severity scores (e.g., Child–Pugh or MELD), frailty assessments, and sarcopenia—were not systematically assessed and therefore could not be included in the multivariable analyses. Consequently, these unmeasured variables may be sources of residual confounding factors. Further studies with larger sample sizes and more comprehensively characterized cohorts are needed to confirm and extend these findings.

## 6. Conclusions

The study findings show that there is a relationship between the cognitive function level and static balance and fatigue severity in patients with liver cirrhosis. However, these findings extend the existing body of literature on this subject by demonstrating the specific associations between cognitive function, balance, and fatigue in patients with cirrhosis. The findings of this study may assist in the formulation of effective strategies to mitigate the severity of fatigue and enhance balance levels in patients suffering from liver cirrhosis. Patients with liver cirrhosis require continuous health management. During this process, they must make significant efforts to delay liver transplantation and ensure that the disease process improves and progresses day by day. To this end, it emphasizes the need for strategic approaches to increase cognitive function levels and reduce fatigue levels. Furthermore, useful strategies should be developed to regulate balance levels and increase physical activity. At the same time, it is crucial to motivate them to manage the disease process and maintain survival. As cognitive function declines in patients with liver cirrhosis, the risk of related complications and death increases. Motor cognitive exercise programs make an important contribution by teaching non-pharmacological interventions that increase cognitive function, reduce fatigue severity, and increase physical activity levels in patients with liver cirrhosis, and by supporting them in coping with their condition.

## Figures and Tables

**Table 1:** Individual characteristics and homogeneity tests of liver cirrhosis (N = 132).

Individual Characteristics					
Means	(Mean ± Sd.)		Min–Max		
Age	53.15 ± 8.97		35–69		
BMI (kg/cm^2^)	24.38 ± 1.98		20–28		
Disease Duration (months)	38.02 ± 1.56		6–71		
	N	%	MoCA	FSS	IPAQ-SF
Gender					
Female	54	40.9	23.72 ± 1.95	4.51 ± 0.28	1059.4 ± 394.2
Male	78	59.1	23.85 ± 2.12	4.48 ± 0.37	984.0 ± 336.1
			U = 2111, *p* = 0.985	U = 2115, *p* = 0.968	U = 2933.500, *p* = 0.364
Educational status					
Elementary school	58	43.9	23.95 ± 2.24	4.49 ± 0.29	1031.7 ± 397.9
High school	50	37.9	23.86 ± 1.71	4.42 ± 0.37	1028.5 ± 315.7
University	24	18.2	23.29 ± 2.19	4.64 ± 0.31	945.8 ± 364.9
			KW = 2.149, *p* = 0.342	KW = 5.798, *p* = 0.055	KW = 1.221, *p* = 0.543
Marital status					
Single	36	27.3	23.67 ± 2.08	4.47 ± 0.28	995.4 ± 367.7
Married	96	72.7	23.84 ± 2.04	4.50 ± 0.35	1066.9 ± 343.7
			U = 1665, *p* = 0.746	U = 1863, *p* = 0.489	U = 1537.500, *p* = 0.309
Economic status					
Low	27	20.5	24.07 ± 2.94	4.63 ± 0.29	931.8 ± 337.8
Medium	74	56.1	23.80 ± 1.75	4.44 ± 0.33	988.4 ± 366.8
High	31	23.5	23.55 ± 1.76	4.48 ± 0.35	1150.3 ± 342.5
			KW = 0.528, *p* = 0.768	KW = 4.426, *p* = 0.109	KW = 4.826, *p* = 0.090
Smoking status					
Yes	41	31.1	23.56 ± 1.85	4.49 ± 0.39	968.0 ± 375.5
No	91	68.9	23.90 ± 2.12	4.49 ± 0.31	1036.0 ± 355.0
			U = 1753, *p* = 0.577	U = 1851, *p* = 0.943	U = 1712.500, *p* = 0.432
Alcohol Use					
Yes	9	6.8	24.00 ± 4.58	4.63 ± 0.38	1014 ± 404.1
No	90	68.2	23.97 ± 1.77	4.50 ± 0.32	1010 ± 356.4
Quit	33	25	23.27 ± 1.68	4.42 ± 0.35	1027 ± 374.9
			KW = 3.866, *p* = 0.145	KW = 2.158, *p* = 0.340	KW = 0.167, *p* = 0.920
Exercise Habits					
Regularly	15	11.4	22.80 ± 2.39	4.64 ± 0.42	1071.3 ± 281.4
Irregularly	45	34.1	24.33 ± 1.50	4.43 ± 0.23	1044.1 ± 429.6
Not Exercising	72	54.5	23.67 ± 2.18	4.50 ± 0.36	984.8 ± 330.0
			KW = 9.290, * ***p* = 0.010**	KW = 6.457, * ***p* = 0.040**	KW = 1.025, *p* = 0.599

MoCA—Montreal cognitive assessment scale; FSS—fatigue severity scale; IPAQ—international physical activity questionnaire (MET·min/week); KW—Kruskal–Wallis test statistic; *p*—level of statistical significance; U—Mann–Whitney test statistic; * *p* < 0.05.

**Table 2:** Patients’ cognitive function, balance, fatigue severity, and physical activity mean scores.

	Min–Max	Mean ± SD
MoCA Total Score	20–30	23.79 ± 2.04
Balance		
Timed Up and Go Test (sec)	7–13	10.36 ± 1.61
One-Legged Stance Test (sec)	8–32	15.63 ± 5.39
FSS Total Score	3.56–5.11	4.49 ± 0.33
IPAQ-SF Total Score	481.10–1582.60	1014.85 ± 361.42

**Table 3:** The relationship between patients’ cognitive function, balance, fatigue severity, and physical activity.

	Timed Up and Go Test	One-Legged Stance Test	FSS	IPAQ-SF
MoCA	r: −0.068 *p*: 0.441	r: 0.232 * *p*: 0.007	r: −0.297 * *p*: 0.001	r: −0.011 *p*: 0.903

r, Spearman’s correlation; * *p* < 0.05.

**Table 4:** Multivariable regression analyses predicting cognitive function.

Predictor	β* (95% CI)	*p*
Age	0.00 (−0.03, 0.03)	0.937
Sex	−0.01 (−0.72, 0.58)	0.828
Disease duration	0.19 (0.00, 0.04)	0.020
One-Legged Stance	0.25 (0.03, 0.16)	0.004
FSS	−0.37 (−0.36, 0.14)	<0.001
IPAQ	0.08 (−0.25, 0.87)	0.273
**Adj, R^2^ = 0.195; F(6125) = 6.29, *p* < 0.001**		

β*—standardised regression coefficient. CI—95% confidence interval. FSS—fatigue severity scale; IPAQ—international physical activity questionnaire.

## Data Availability

The original contributions presented in this study are included in the article. Further inquiries can be directed to the corresponding authors.
